# Piwi-Interacting RNAs: A New Class of Regulator in Human Breast Cancer

**DOI:** 10.3389/fonc.2021.695077

**Published:** 2021-07-06

**Authors:** Lu Qian, Heying Xie, Libo Zhang, Qian Zhao, Jinhui Lü, Zuoren Yu

**Affiliations:** ^1^ Research Center for Translational Medicine, Shanghai East Hospital, Tongji University School of Medicine, Shanghai, China; ^2^ Jinzhou Medical University, School of Basic Medical Sciences, Jinzhou, China

**Keywords:** piRNA, tumorigenesis, breast cancer, cancer stem cell, PIWI

## Abstract

P-element-induced wimpy testis (Piwi)-interacting RNAs (piRNAs) are a class of germline-enriched small non-coding RNA that associate with Piwi family proteins and mostly induce transposon silencing and epigenetic regulation. Emerging evidence indicated the aberrant expression of Piwil proteins and associated piRNAs in multiple types of human cancer including breast cancer. Although the majority of piRNAs in breast cancer remains unclear of the function mainly due to the variety of regulatory mechanisms, the potential of piRNAs serving as biomarkers for cancer diagnosis and prognosis or therapeutic targets for cancer treatment has been demonstrated by *in vitro* and *in vivo* studies. Herein we summarized the research progress of oncogenic or tumor suppressing piRNAs and their regulatory mechanisms in regulating human breast cancer, including piR-021285, piR-823, piR-932, piR-36712, piR-016658, piR-016975 and piR-4987. The challenges and perspectives of piRNAs in the field of human cancer were discussed.

## Introduction

P-element-induced wimpy testis (Piwi)-interacting RNAs (piRNAs) are a class of endogenous non-coding RNA with 26-32nt in length that associate with Piwi family proteins and are specifically expressed and enriched in mammalian germ cells (1). Although piRNA sequences were originally discovered from the repetitive genomic elements of D. melanogaster germline in 2001 by Aravin et al. (2), until 2006 a class of highly abundant small RNAs around 30 nt in length were identified by Girard et al. in mammalian testes, binding to MIWI, a murine Piwi protein, and thereby was named piRNA (3). piRNAs can be derived from transposons, mRNAs or long non-coding RNAs (lncRNAs). Up to date, thousands of piRNA sequences have been identified in germ cells. However, their function mostly remains unknown (4).

Literature has revealed the complexity of the biogenesis of piRNAs, in which a vast amount of proteins are involved (2, 3, 5). There are two approaches have been well demonstrated for the biogenesis of piRNAs including primary biogenesis from cluster transcripts and secondary biogenesis from the ping-pong cycle (5). After transcription from genomic loci carrying transposon fragments, the cluster transcripts are spliced into piRNA precursors (pre-piRNAs), followed by the transit from the inner side to the outer side of the nuclear pore associated with some ribonucleic proteins, such as Nuclear Export Factor 2 (Nxf2) and its co-factor Nuclear Transport Factor 2-like Export Factor 1 (Nxt1) (6). Then pre-piRNAs are either processed through Zucchini (Zuc)-dependent mechanism with Piwi protein involved, or processed through a ping-pong cycle with Aubergine (Aub) and Argonaute3 (Ago3) involved (5). Notably, processing of pre-piRNAs usually takes place within a specialized subcellular perinuclear structure, termed nuage in mammals and Yb bodies in Drosophila, locating around the mitochondria in the cytoplasm (6, 7). Vret, as one of the most important proteins in both Yb bodies and nuage, has been shown to be required for generation of Piwi-bound piRNAs and localization of Piwi proteins (8).

In germline, a few of piRNAs have been demonstrated to play important roles in regulating germ stem cell maintenance, spermatogenesis, meiosis, transposon silencing, genome rearrangement, and genomic integrity by piRNA-induced silencing complexes (1, 3). In addition, some piRNAs were reported to regulate heterochromatin formation, DNA methylation, and gene expression at transcriptional or post-transcriptional levels (9, 10). In particular, those piRNAs derived from pseudogenes or antisense transcripts usually show regulation of the corresponding endogenous genes (4).

## Aberrant Expression of piRNAs and Piwi Members in Cancer

In addition to the enrichment of piRNAs in germ cells, emerging evidence indicates the existence of piRNAs in the somatic stem cells and tumor cells (11, 12). Connections between piRNA/Piwi complex and tumorigenesis have been frequently reported (12–14). Aberrant expression of piRNAs and Piwi proteins in cancer cells may be an indication of the involvement of piRNAs in the regulation of cancer development and progression.

The Ago/Piwi family is not only required for germline development, but also plays central roles in transcriptional and posttranscriptional gene regulation and transposon silencing mediated by piRNAs. In D. melanogaster, the Piwi family consists of Piwi, Aub and Ago3. In the human testis, four homologues Hiwi, Hili, Hiwi2, and Piwil3 have been identified as Piwi family members. Their function in germline has been implicated, but the somatic function remains unclear.

Martinez et al. analyzed 6,260 human piRNA transcriptomes derived from non-malignant and tumor tissues of 11 organs, and discovered 273 and 522 piRNAs with expression in somatic non-malignant tissues and corresponding tumor tissues, respectively, which were able to not only distinguish tissue-of-origin, but also distinguish tumors from non-malignant tissues in a cancer-type specific manner (15). A recent study identified the increased levels of circulating miR-1307-3p, piR-019308, piR-004918 and piR-018569 in the serum exosomes of gastric cancer patients (16). Moreover, higher levels of piR-004918 and piR-019308 were found in the gastric cancer patients with metastasis than non-metastatic patients, indicating that circulating piRNAs in serum are promising non-invasive diagnostic biomarkers for gastric cancer patients and potential markers for monitoring metastasis (16). Wang et al. screened piRNAs in the serum from 7 patients with colorectal cancer and 7 normal controls using small RNA sequencing, followed by QRT-PCR validation of the differentially expressed piRNAs in a training cohort of 140 patients with colorectal cancer (17). As a result, piR-020619 and piR-020450 showed upregulation in the serum of patients. Meanwhile, their expression was also analyzed in 50 patients with lung cancer, 50 with breast cancer, and 50 with gastric cancer, but did not show change in serum of patients with lung, breast, and gastric cancer, indicating the specificity of piR-020619 and piR-020450 as biomarkers for early detection of colorectal cancer (17).

piRNAs also showed regulation by transcriptional factors in human cancer. For example, upregulation of piR-34871 and piR-52200 and downregulation of piR-35127 and piR-46545 by the Ras Association Domain Family Member 1C (RASSF1C) were reported in the lung cancer cell line H1299 and lung tumor tissues (18). Overexpression of piR-35127 and piR-46545 and knockdown of piR-34871 and piR-52200 significantly reduced cell proliferation in both lung cancer cell lines (A549 and H1299) and breast cancer cell lines (Hs578T and MDA-MB-231) (18). Notably, RASSF1C, known as an oncogene, was reported to regulate Piwil1 in lung cancer (19). In MDA-MB-231 breast cancer cells, “cancer-testis gene” Glycerol-3-phosphate acyltransferase-2 (GPAT2) showed regulation on the expression of piRNAs and tRNA-derived fragments. The most GPAT2-regulated piRNAs are single copy in the genome and previously found to be upregulated in cancer cells (20).

Although Piwi-like (Piwil) genes including Piwil1, Piwil2, Piwil3, and Piwil4 have been detected in various types of cancer tumors, such as renal cell carcinoma (21) and breast cancer (22), overexpression of Piwil1 was the most frequently reported in cancers including lung cancer (23), gastric cancer (24), renal cancer (25) and colorectal cancer (26), in which high levels of Piwil1 showed significant correlation with short survival and/or poor prognosis in the patients with cancers (23–26). Piwil2 is highly expressed in glioma, and correlates with poor patient prognosis. Piwil2 plays an important role in the transformation of cervical epithelial cells to tumor-initiating cells by epigenetic regulation (27). In human breast cancer, Piwil2 overexpression was frequently reported to associate with piRNAs (28–30), functioning as an oncogene. Piwil3 showed a tumor-type dependent function as an oncogene or tumor suppressor. In glioma tissues, Piwil3 is downregulated, and negatively associated with pathological grade (31). In gastric cancer, overexpression of Piwil3 promoted cell proliferation, migration, and invasion *via* the JAK2/STAT3 signaling pathway (32). In malignant melanoma, upregulation of Piwil3 was association with cancer aggressiveness and progression (33). For Piwil4, Wang et al. reported high expression of Piwil4 in both breast cancer tissues and MDA-MB-231 breast cancer cell line. Knockdown of Piwil4 in MDA-MB-231 cells dramatically suppressed the cell migration and proliferation through regulating TGF-β and FGF signaling pathways and MHC class II proteins (34). In hepatocellular carcinoma, co-expression of Piwil2/Piwil4 has potential as an indicator for tumor prognosis (35).

## Regulatory Function of piRNAs and Piwi Members in Cancer

Although the oncogenic function of Piwil1 in gastric cancer cells was reported to be independent of its partner piRNAs (36), Piwi proteins have showed significant synergy with related piRNAs in regulating human cancer (28, 29). Complex formed by piRNAs binding to Piwi proteins, such as piR-651/Piwil 1 in gastric cancer (24), piR-54265/Piwil 2 in colorectal cancer (37), piR-932/Piwil 2 in breast cancer (29), have been demonstrated to regulate cell proliferation, invasion and metastasis of cancer cells.

Overexpression of piR-651 was reported in gastric cancer compared to normal control tissues. Gastric cancer cells were arrested at G2/M phase after knockdown of piR-651 (12). Upregulation of piR-823 and its oncogenic function were reported in both esophageal cancer and breast cancer (38, 39). In the luminal subtype of breast cancer cells, overexpression of piR-823 increased the expression of DNA methyltransferase DNMT1, DNMT3A, and DNMT3B, promoted DNA methylation of gene adenomatous polyposis coli (APC), thereby activating Wnt signaling and inducing cancer cell stemness (38). In esophageal squamous cell carcinoma, piR-823 showed significantly upregulation in tumor tissues, compared with matched normal control. The level of piR-823 was significantly associated with lymph node metastasis. In addition, a positive correlation between piR-823 and DNMT3B expression was observed in esophageal cancer (39). However, a recent publication showed tumor repression function of piR-823 in gastric cancer (40), indicating the complexity of the piRNA functions in a cancer type-dependent manner.

In addition to the epigenetic regulation of piRNAs in control of cancer development and progression (15, 22, 38), piRNAs involve in cancer regulation by altering the expression of cancer-related genes in a mechanism similar to microRNA (miRNA). For example, in lung cancer cells piR-55490 was demonstrated to bind with 3′UTR of mTOR mRNA and induce its degradation (41). Like the “seed sequence” of miRNAs, the 5’ end of piR-55490 can be complementary to the 3’UTR of mTOR mRNA. In addition, piR-55490 was found to be silenced in lung carcinoma specimens and cell lines, compared with normal controls. Moreover, the expression level of piR-55490 showed a negatively correlation with patients’ survival. Restoration of piR-55490 suppressed cell proliferation in lung cancer by suppressing Akt/mTOR pathway (41).

Overall, evidence demonstrating the potential clinical significance of piRNA and Piwi proteins as diagnostic tools, therapeutic targets, and/or prognosis biomarkers in cancer is increasing. The relevant progress in cancer has been recently reviewed (42, 43). Herein, we highlighted the research progress and clinical potential of piRNAs in human breast cancer.

## piRNAs in Breast Cancer

Breast cancer is the most common cancer and the leading cause of cancer-related death in women all over the world. Abnormal expression of some piRNAs has been reported in breast cancer cells and tissues, showing relevance to the tumor development and progression (**Table 1**). Those aberrantly expressed piRNAs in breast cancer including piR-4987, piR-021285, piR-823, piR-932, piR-36712, piR-016658 and piR-016975 may held potential to be developed as biomarkers and/or therapeutic targets in breast cancer. Our recent study also identified 415 piRNA sequences from the medium of luminal subtype of human breast cancer cell MCF-7, in which 27 piRNAs showed deregulation by pro-oncogene cyclin D1 (28). Huang et al. reported 4 piRNAs including piR-4987, piR-20365, piR-20485 and piR-20582 with upregulation in tumor tissues compared with matched non-tumor tissues in 50 patients with breast cancer (14). Moreover, upregulation of piR-4987 showed association with lymph node positivity (14). Hashim et al. identified over 100 piRNAs in breast cancer cell lines and tumor biopsies by a small RNA-Seq analysis (48). A mRNA targets analysis using piRNome tool further revealed 10 piRNAs with a specific expression pattern in breast tumors targeting key cancer cell pathways (48), in which DQ596670, DQ598183, DQ597341, DQ598252, and DQ596311 were downregulated, while DQ598677, DQ597960, and DQ570994 were upregulated in tumor tissues of breast cancer. Park et al. and Lee et al. applied molecular beacons (a synthetic structured DNA oligonucleotide probe) to detect piR-36026 and/or piR-36743 in a single breast cancer cell, showing their different expression in a cancer cell subtype-dependent manner (49, 50). Kärkkäinen et al. performed small RNA sequencing analysis in 227 fresh-frozen breast tissue samples (51). Three small RNAs annotated as piRNA database entries (DQ596932, DQ570994, and DQ571955) were detected in the tumor samples, all showing upregulation in grade III tumors. Furthermore, patients with estrogen receptor positive and DQ571955 high had shorter relapse-free survival, suggesting DQ571955 as a potential marker for predicting radiotherapy response in estrogen receptor positive breast cancer. In addition, DQ570994 showed potential for predicting tamoxifen and chemotherapy response (51). In triple negative breast cancer (TNBC), neoadjuvant chemotherapy (NACT) is increasingly applied to the therapy management due to its positive association with response rates of patients. A recent work reported the potential of circulating piR−36743, miR−17, −19b and −30b as diagnostic biomarkers in the monitoring of NACT−driven complete clinical response in TNBC (52). Koduru et al. used public database (small RNA sequencing data) derived from 24 TNBC tumors and 14 adjacent normal tissue samples, and identified a group of differentially expressed miRNAs, piRNAs, lncRNAs and sn/snoRNAs. The top five upregulated piRNAs in tumor tissues are piR-21131, -32745, -21131, -1282, -23672, and top five downregulated piRNAs are piR-23662, -26526, -26527, -30293 and -26528 (53).

**Table 1:** Deregulated piRNAs in human breast cancer.

piRNA ID	up/down	Regulatory Function	Mechanism	Reference
piR-021285	up	Promoting invasiveness	DNA methylation	(44)
piRNA-823	up	Promoting cancer cell stemness	DNA methylation/Wnt Signaling	(38)
piRNA-932	up	Promoting EMT and metastasis	DNA methylation	(29)
piR-016658	up	Upregulated in cancer stem cells	N/A	(28)
piR-651	up	N/A	Estrogen regulation	(45)
piR-4987	up	Correlation with lymph node positivity	N/A	(14)
piR-20365, piR-20485, piR-20582	up	N/A	N/A	(14)
piR-36712	down	Suppressing EMT/Related with chemoresistance	Competing endogenous RNAs	(46)
piR-016975	down	Downregulated in cancer stem cell	N/A	(28)
piR-FTH1	down	Promoting chemosensitivity	Targeting FTH1 mRNA	(47)

N/A, not available.

Estrogen signaling has been well demonstrated to play important roles in tumor development and progression of breast cancer through interacting with two receptors ERα and ERβ. ERα, as a predominant endocrine regulatory protein in estrogen‐induced breast cancer, interacts with non-coding RNAs including piRNAs (54). However, the interaction between ERβ and non-coding RNAs in breast cancer remains unclear. Alexandrova et al. performed sncRNA sequencing analysis on ERβ-expressing TNBC cell lines and 12 ERβ+ and 32 ERβ− TNBC tissue samples (55). A group of ERβ-regulated small ncRNAs was identified in TNBC, including miR-181a-5p and piR-31143 with aberrant upregulation in the ERβ+ tumor samples (55)

Although hundreds of piRNAs have been identified in human breast cancer, only a few of them including piR-021285, piR-823, piR-932, piR-36712, piR-016658 and piR-016975 were determined with the regulatory function and molecular mechanisms, as described in detail below.

### piR-021285 in Breast Cancer

piR-021285 is the first piRNA showing regulation of human tumorigenesis in breast cancer *via* an epigenetic mechanism of DNA methylation (44). A genome-wide methylation screening analysis in the piR-021285 mimic-transfected MCF7 cells revealed significant induction of DNA methylation in a number of breast cancer-related genes including ARHGAP11A, which was associated with increased transcription of ARHGAP11A and enhanced invasiveness of MCF-7 cells (44). The expression and/or function of piR-021285 in other types of cancer have yet to be determined.

### piRNA-823 in Breast Cancer

piR-823 is another piRNA having regulatory function in human breast cancer through epigenetic mechanism. The carcinogenicity of piR-823 in malignant breast cancer may be related with estrogen status since external administration of estrogen increased piR–823 expression in estrogen receptor negative MDA-MB–231 cells, while reduced the piR–823 levels in estrogen receptor positive MCF-7 cells (45). Further study demonstrated oncogenic function of piR-823 in estrogen receptor positive luminal subtype of breast cancer *via* regulating cancer cell stemness mediated by altered DNA methylation and activated Wnt signaling (38).

In comparison with breast cancer, several other cancer types also showed regulation by piR-823. For example, Increased piRNA-823 expression was associated with late stages and poor prognosis of multiple myeloma. piRNA-823 in the multiple myeloma-derived extracellular vesicles was demonstrated to promote tumorigenesis through re-educating endothelial cells in the tumor microenvironment (56). In colorectal cancer, piRNA-823 increased cell proliferation, invasion and apoptosis resistance through inhibiting the ubiquitination of HIF-1α, thereby upregulating the glucose consumption of cancer cells and inhibiting intracellular reactive oxygen species (57).

### piR-932 in Breast Cancer

In addition to piR-021285 and piR-823, piR-932 also showed overexpression in human breast cancer, playing tumor-promoting roles through epigenetic mechanism (29). In Piwil2 positive breast cancer stem cells, piR-932 could bind with Piwil2 to suppress the expression of Latexin by promoting methylation of the CpG island at its promoter region. In view of the negative regulation of cancer stem cells by Latexin (58), piR-932/Piwil2 could be the potential targets for suppressing the progression of breast cancer. Up to date, piR-932 has not been reported in other types of cancer.

### piR-36712 in Breast Cancer

piR-36712 was reported having a lower expression level in breast cancer tumors than that in normal tissues, functioning as a tumor suppressor but dependent on the expression of SEPW1 and p53 (46). piRNA-36712 inhibited SEPW1 through interacting with its pseudogene SEPW1P, which thereafter suppressed p53, leading to the upregulation of Slug but downregulation of p21 and E-cadherin, thus performing oncogenic function in human breast cancer. In addition, piR-36712 showed involvement in chemo-sensitivity of breast cancer cells in response to paclitaxel or doxorubicin (46). There is still no literature about piR-36712 study in other cancers.

### piR-016658 and piR-016975 in Breast Cancer

Our previous study found upregulation of piR-016658 and downregulation of piR-016975 by cyclin D1 in human breast cancer (28). Further analysis indicated the correlation of piR-016658 and piR-016975 with breast cancer cell stemness. High levels of piR-016658 were found in the basal-like breast cancer cells, as well as Aldehyde dehydrogenase 1 (ALDH1) positive breast cancer stem cells isolated from breast cancer tumors. In contrast, lower levels of piR-016975 were determined not only in basal-like subtype of breast cancer cells compared to luminal subtype, but also in breast cancer stem cells compared to non-stem breast cancer cells. In view of the germ stem cell maintaining function of piRNAs in germline, this study demonstrated that piRNAs are able to regulate cell stemness characteristics in human breast cancer, thereby regulating tumor growth and progression.

### piR-FTH1 in Breast Cancer

Ferritin heavy chain (FTH1), as a key regulator of iron metabolism, was recently identified as a favorable prognostic gene for patients with TNBC. Fth1 levels are associated with the progression of breast cancer and chemo-sensitivity of breast cancer cells (59). Balaratnam et al. discovered a human piRNA piR-FTH1 with sequence complementary to Fth1 mRNA in human somatic cells. In the tested cancer cells, piR-FTH1 and Fth1 showed inverse correlation in expression. In addition, piR-FTH1 can downregulate the Fth1 expression at post-transcriptional level in TNBC cells *via* a HIWI2/HILI mediated mechanism (47).

## Future Perspective

It has been widely acknowledged that the cell of origin of cancer is a deregulated somatic cell that loses normal regulatory mechanism and reproduces itself without control. The primitive cancer life cycle contains primary cancer stem cells, somatic cells and reproductive cells (60). In view of the similarity between cancer stem cells and germ cells of stemness properties and reproductive ability, there are some regulatory mechanisms in common shared by the two cell types. Although piRNAs were originally identified as a group of germline-specific non-coding small RNAs, emerging evidence revealed their aberrant expression in human cancers. Functional assays indicated the regulation of cancer cell proliferation, EMT, metastasis and cancer stem cells by piRNAs. Overall, piRNAs may have potential to be developed as biomarkers for cancer diagnosis and prognosis, and/or as therapeutic targets for cancer treatment.

Although the studies of piRNAs and Piwi family members in cancer are adding a novel page in the history of cancer research, currently the majority of piRNAs remains unclear of their function in regulating human cancer. There are several key challenges about piRNAs that we are facing, and need to solve, including 1) whether biogenesis of piRNAs in cancer cells shares similar approaches with that in germline; 2) what is the main regulatory mechanism of piRNAs in control of cancer development and progression; 3) how to predict the target DNAs of piRNAs when epigenetically regulating gene expression. It is hoped that with these challenges addressed and the regulatory mechanisms revealed, along with the application of piRNAs in suppression of tumorigenesis and cancer progression in the animal models, piRNAs and Piwi proteins may lead to novel therapeutic approaches in treatment of cancer, including breast cancer.

## Author Contributions

LQ, HX, LZ, QZ, and JL wrote a part of the draft. ZY designed the work and revised the draft. All authors contributed to the article and approved the submitted version.

## Funding

This work was supported by grant from the National Key Research and Development Program of China Stem Cell and Translational Research (2016YFA0101202); grants 81772810 and 81972476 (YZ), 82002789 (JL) from the National Natural Science Foundation of China; Grant 20JC1410400 from Science and Technology Commission of Shanghai Municipality; grant 19DZ2251000 from Shanghai Engineering Research Center of Artificial Heart and Heart Failure.

## Conflict of Interest

The authors declare that the research was conducted in the absence of any commercial or financial relationships that could be construed as a potential conflict of interest.
